# Particulate matter causes skin barrier dysfunction

**DOI:** 10.1172/jci.insight.145185

**Published:** 2021-03-08

**Authors:** Byung Eui Kim, Jihyun Kim, Elena Goleva, Evgeny Berdyshev, Jinyoung Lee, Kathryn A. Vang, Un Ha Lee, SongYi Han, Susan Leung, Clifton F. Hall, Na-Rae Kim, Irina Bronova, Eu Jin Lee, Hye-Ran Yang, Donald Y.M. Leung, Kangmo Ahn

**Affiliations:** 1 Department of Pediatrics, National Jewish Health, Denver, Colorado, USA.; 2 Department of Pediatrics, Samsung Medical Center, Sungkyunkwan University School of Medicine, Seoul, South Korea.; 3 Environmental Health Center for Atopic Diseases, Samsung Medical Center, Seoul, South Korea.; 4 Department of Medicine, National Jewish Health, Denver, Colorado, USA.; 5 Department of Dermatology, Sanggye Paik Hospital, Inje University College of Medicine, Seoul, South Korea.; 6 Seoul Metropolitan Government Research Institute of Public Health and Environment, Seoul, South Korea.

**Keywords:** Dermatology, Inflammation, Mouse models, Skin

## Abstract

The molecular mechanisms that underlie the detrimental effects of particulate matter (PM) on skin barrier function are poorly understood. In this study, the effects of PM_2.5_ on filaggrin (FLG) and skin barrier function were investigated in vitro and in vivo. The levels of FLG degradation products, including pyrrolidone carboxylic acid, urocanic acid (UCA), and *cis*/*trans*-UCA, were significantly decreased in skin tape stripping samples of study subjects when they moved from Denver, an area with low PM_2.5_, to Seoul, an area with high PM_2.5_ count. Experimentally, PM_2.5_ collected in Seoul inhibited FLG, loricrin, keratin-1, desmocollin-1, and corneodesmosin but did not modulate involucrin or claudin-1 in keratinocyte cultures. Moreover, FLG protein expression was inhibited in human skin equivalents and murine skin treated with PM_2.5_. We demonstrate that this process was mediated by PM_2.5_-induced TNF-α and was aryl hydrocarbon receptor dependent. PM_2.5_ exposure compromised skin barrier function, resulting in increased transepidermal water loss, and enhanced the penetration of FITC-dextran in organotypic and mouse skin. PM_2.5_-induced TNF-α caused FLG deficiency in the skin and subsequently induced skin barrier dysfunction. Compromised skin barrier due to PM_2.5_ exposure may contribute to the development and the exacerbation of allergic diseases such as atopic dermatitis.

## Graphical abstract

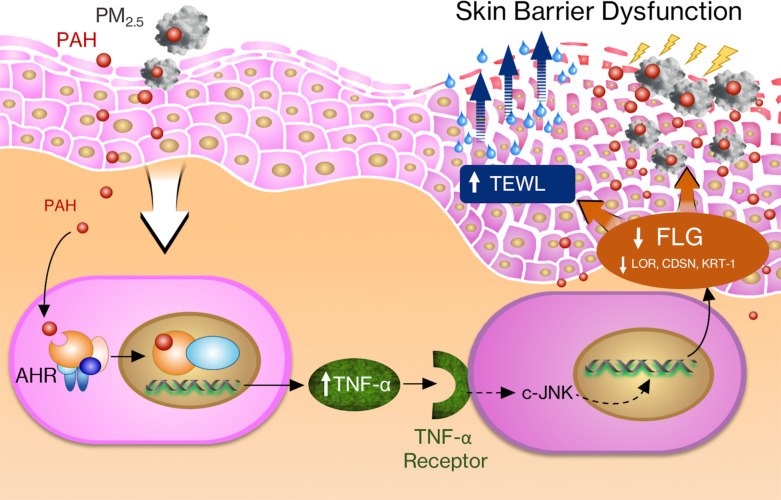

## Introduction

Particulate matter (PM) is one of the major air pollutants and a major health concern that continues to grow with industrialization and urbanization (1–3). The impact of PM is notable, given its socioeconomic burden and the fact that about 9 million people die of PM-associated diseases per year worldwide (3–5). PM is classified on the basis of its aerodynamic diameter: PM_10_ (<10 μm), PM_2.5_ (<2.5 μm), and PM_0.1_ (<0.1 μm) (1, 6). The majority of the particle mass is in the fraction with less than 2.5 μm, and these particles can carry a large amount of absorbed pollutants, oxidants, and organic compounds (6). Jin et al. reported that PM penetrates the epidermis through hair follicles in normal, intact skin and causes cutaneous inflammation in a mouse model (7). Furthermore, it has been reported that polycyclic aromatic hydrocarbons (PAHs), major components of PM_2.5_, easily penetrate the skin in animal models because of their lipophilic nature (1, 8–10). At the molecular level, PAHs have been shown to activate aryl hydrocarbon receptors (AHRs) while binding to the AHR in cytoplasm, then induce translocation of AHR to the cell nuclei to regulate cellular gene expression (11, 12).

The epidermis, the outermost part of the skin, provides a physical and functional barrier to prevent invasions of allergens, pathogens, and air pollutants such as PM into the human body (13–15). Recently, it has been suggested that a disrupted skin barrier promotes epicutaneous sensitization (14, 16). Epithelial barrier dysfunction induces type 2 immune responses and is considered an initial step in developing atopic dermatitis (AD) and the atopic march (14, 16, 17). Epidermal barrier proteins such as filaggrin (FLG) play pivotal roles in maintaining normal skin barrier function (13, 18, 19). FLG degradation products (FDPs) including pyrrolidone carboxylic acid (PCA) and urocanic acid (UCA) are essential for the regulation of skin hydration, pH, photoprotection, and normal epidermal barrier function (19, 20). Recent meta-analysis (21) and epidemiologic studies (22, 23) showed PM_2.5_ is significantly associated with the development and the exacerbation of skin diseases such as AD. Our previous epidemiologic studies also showed that PM_2.5_ is associated with the exacerbation of AD in children (24–26). Therefore, we hypothesized that PM_2.5_ causes FLG deficiency and skin barrier dysfunction.

Most published papers related to PM_2.5_ have focused on cardiovascular and airway diseases, with very few studies examining the effects of PM_2.5_ in the skin. To date, limited and conflicting data regarding the effects of PM on skin and skin barrier proteins have been reported (27–29). Given the importance of potential detrimental effects of PM_2.5_ skin exposure, this study was done to evaluate the effects of PM_2.5_ on skin barrier function using in vitro and in vivo models. Doses of PM_2.5_ chosen for this study reflect physiologic concentrations of ambient PM_2.5_ in the polluted urban area environment. In this study, we present that PM_2.5_-induced TNF-α causes FLG deficiency via AHR and results in subsequent skin barrier dysfunction.

## Results

### Skin FDP levels are decreased in subjects who live in a high-PM_2.5_ environment.

**Figure 1:**
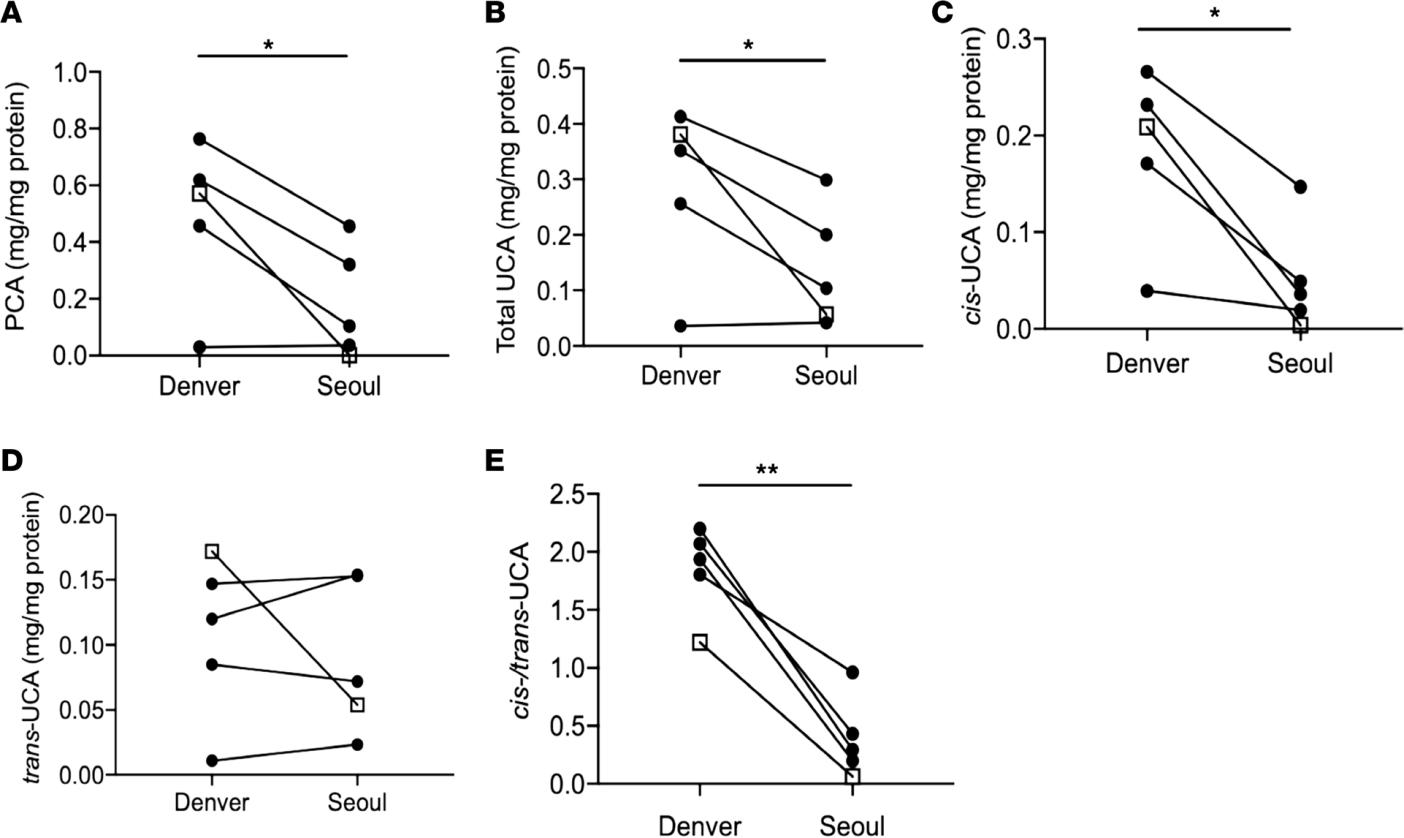
FDP levels decrease in the same subjects after moving from Denver, USA, to Seoul, South Korea, during the same winter season. STS samples were collected from 4 normal, healthy subjects and 1 convalescent AD subject. A total of 10 consecutive STS samples were collected from the same subjects several months apart while they lived in Denver and Seoul. STS samples were processed, and FDP levels were evaluated using liquid chromatography electrospray ionization tandem mass spectrometry (LC-ESI-MS/MS). (**A**) PCA. (**B**) Total UCA. (**C**) *Cis*-UCA. (**D**) *Trans*-UCA. (**E**) *Cis/trans*-UCA. Circles, normal healthy subjects; squares, convalescent AD subjects. *n* = 5 per group. *P < 0.05, **P < 0.01 by paired 2-tailed Student’s *t* test.

During this study, Seoul had PM_2.5_ levels of 100~180 μg/m^3^ and Denver had PM_2.5_ levels of 25~35 μg/m^3^ (Real-time Air Quality Index, https://aqicn.org; AirNow, https://www.airnow.gov). The major components of PAHs in Seoul and Denver were similar, and the average levels of PAHs during the winter season, according to the published data, are 16.1 ± 10.1ng/m^3^ and 3.1 ± 0.4 ng/m^3^ in Seoul and Denver, respectively (30–32). This shows that the levels of PM_2.5_ and PAHs were about 5 times higher in Seoul, Korea, as compared with Denver, Colorado, USA. In this study, skin tape stripping (STS) samples were collected from the same subjects while they lived in Denver and when they subsequently moved to Seoul during the same winter season. STS samples were analyzed for FDPs including PCA and UCA, as these FDPs reflect the level of FLG in the skin (33). As shown in Figure 1, the levels of PCA (Figure 1A, *P* < 0.05), total UCA (Figure 1B, *P* < 0.05), *cis*-UCA (Figure 1C, *P* < 0.05), and *cis/trans*-UCA (Figure 1E, *P* < 0.01) were significantly decreased in STS samples from Seoul compared to those from Denver in the same subjects. However, no difference was noted in *trans*-UCA (Figure 1D). In addition, the clinical rash in a subject who had a history of severe AD in Seoul had cleared after moving to Denver.

### PM_2.5_ inhibits FLG expression and increases transepidermal water loss.

**Figure 2:**
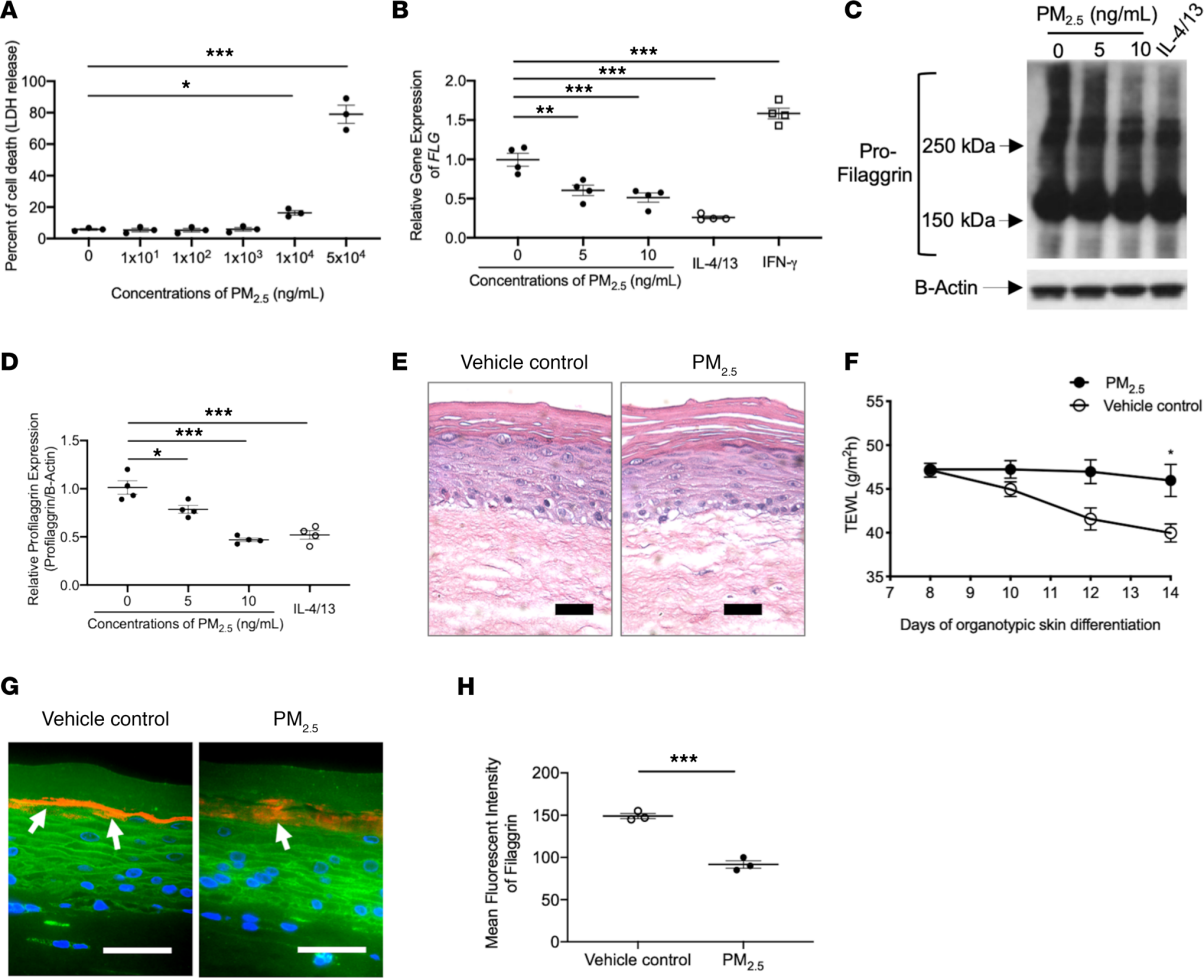
Effects of PM_2.5_ on FLG and skin barrier function in cultured keratinocytes and organotypic skin. (**A**) The percentage of cell death (lactate dehydrogenase release into cell culture media) is increased after exposure to PM_2.5_. Gene (**B**) and protein (**C** and **D**) expressions of FLG in cultured HEKs were evaluated using reverse transcriptase PCR (RT-PCR) and Western blotting, respectively, and demonstrated reduced *FLG* mRNA and protein expression in PM_2.5_-treated cultures. H&E staining (**E**) and TEWL (**F**) in organotypic skin. FLG protein expression (**G** and **H**) was evaluated in organotypic skin using immunofluorescence staining. Arrows point to FLG staining (shown in red). Wheat germ agglutinin–conjugated FITC (green) was used to stain the cytoskeleton. Nuclei were visualized with DAPI (blue). Data are representative of 3 independent experimental repetitions using 3 different lots of HEKs. The data are shown as the mean ± SEM. *n* = 3–4 per group. Scale bar: 50 μm. *P < 0.05, **P < 0.01, ***P < 0.001 by 1-way ANOVA with Tukey-Kramer test (**A**, **B**, and **D**) and 2-tailed Student’s *t* test (**F** and **H**).

To understand the direct relationship between increased PM_2.5_ and FLG breakdown production, we studied human epidermal primary keratinocyte (HEK) cultures in vitro to examine whether exposure to PM_2.5_ can alter keratinocyte expression of FLG. Initially, a cytotoxicity assay was performed to determine optimal sublytic concentrations of PM_2.5_ for experiments. HEKs were differentiated for 3 days and then stimulated with various concentrations of PM_2.5_ for 48 hours. Minimal toxicity (<6% cell death) was noted in cultures stimulated with up to 1000 ng/mL of PM_2.5_ compared with the cells treated with media alone (Figure 2A). However, the percentage of cell death was significantly increased in cells treated with 10 μg/mL (*P* < 0.05) and 50 μg/mL (*P* < 0.01) of PM_2.5_ compared with cells treated with media alone (Figure 2A). Therefore, less than 1000 ng/mL of PM_2.5_ was used for our remaining experiments.

As depicted in Figure 2, gene expression of *FLG* was significantly (*P* < 0.01) decreased in HEKs treated with PM_2.5_ as low as 5 ng/mL compared with cells treated with media alone (Figure 2B). *FLG* expression was inhibited by Th2 cytokines (*P* < 0.001) and upregulated by IFN-γ (*P* < 0.001) (Figure 2B) as shown before (34). These findings were also confirmed at protein levels using Western blotting (Figure 2, C and D). Cytokine modulation of FLG protein by Th2 cytokines and IFN-γ have been reported previously (34). FLG is produced as an FLG polymer (pro-FLG > 400 kDa) and is proteolyzed to monomeric FLG in the cornified epidermis; this process takes 3~4 weeks (20, 35). In the current study, we stimulated differentiated keratinocytes with PM_2.5_ for 2 days and evaluated the FLG expression. At this time, as shown in Figure 2C, the levels of large–molecular weight forms of pro-FLG (>150 kDa) were decreased by PM_2.5_ treatment, but the smaller molecular weight FLG products (<150 kDa) were less affected by PM_2.5_ treatment, likely due to the insufficient time for the full proteolytic processing of the pro-FLG after PM_2.5_ treatment. PM_2.5_ also inhibited gene expression of loricrin (*LOR*), keratin-1, desmocollin-1, and corneodesmosin (Supplemental Figure 1; supplemental material available online with this article; https://doi.org/10.1172/jci.insight.145185DS1) in keratinocytes. However, the gene expression of involucrin (*IVL*) and claudin-1 in keratinocytes was not affected by PM_2.5_ (Supplemental Figure 1).

To further evaluate PM_2.5_-mediated inhibition of FLG and epidermal barrier function, 3-dimensional organotypic skin cultures were generated and differentiated for 7 days, followed by treatment with a vehicle or PM_2.5_ (1 ng/mL) for an additional 7 days. Transepidermal water loss (TEWL) and FLG expression were evaluated. In H&E staining, the cornified layer of organotypic skin culture treated with PM_2.5_ was less differentiated compared with that of skin treated with vehicle (Figure 2E), and TEWL was significantly (*P* < 0.05) higher in organotypic skin cultures treated with PM_2.5_ as compared with skin treated with vehicle (Figure 2F). Additionally, the staining intensity of FLG was significantly (*P* < 0.001) decreased in organotypic skin treated with PM_2.5_ compared with skin treated with vehicle control (Figure 2, G and H). These findings suggest that PM_2.5_ can cause FLG deficiency and epidermal barrier dysfunction.

### PM_2.5_ induces expression of AHR and causes nuclear translocation of AHR.

**Figure 3:**
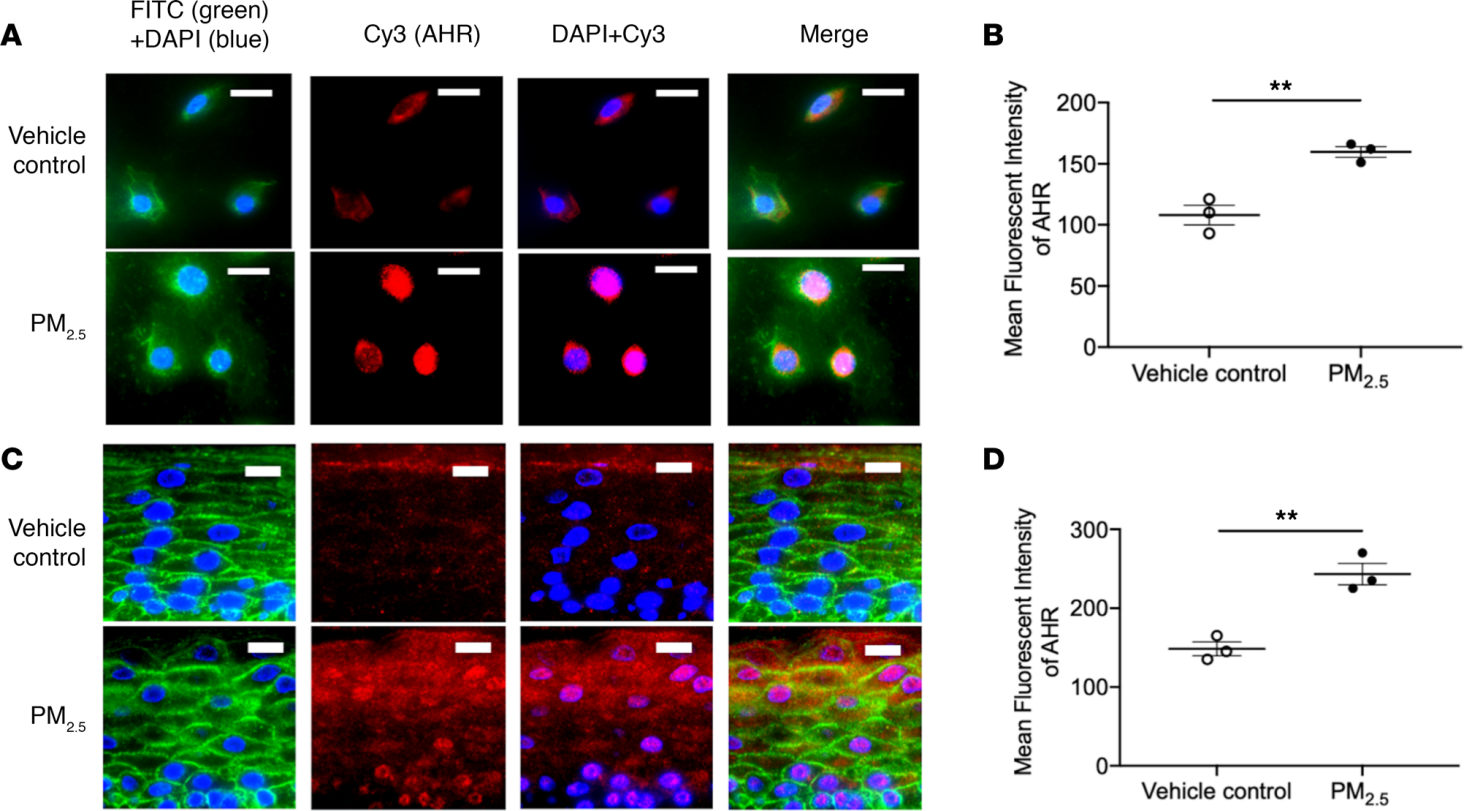
Effect of PM_2.5_ on AHR in both human primary keratinocytes and organotypic skin. Expressions of AHR (red) in both cultured HEKs (**A** and **B**) and organotypic skin (**C** and **D**) were evaluated using immunofluorescence staining and demonstrated a reduction in FLG expression after PM_2.5_ exposure. Wheat germ agglutinin–conjugated FITC (green) was used to stain the cytoskeleton. Nuclei were visualized with DAPI (blue). Data are representative of 3 independent experimental repetitions. The data are shown as the mean ± SEM. *n* = 3 per group. Scale bar: 50 μm. **P < 0.01 by 2-tailed Student’s *t* test.

It has been reported that PAHs, a major component of PM_2.5_, induce nuclear translocation of AHR in stimulated cells and modulate gene expression (11, 12). Therefore, we examined whether PM_2.5_-regulated AHR expression in keratinocytes and influenced AHR cellular localization. After 24 hours of treatment with PM_2.5_, AHR was mostly localized in the nuclei of keratinocytes (Figure 3A). The AHR staining intensity was significantly (*P* < 0.01) increased in HEKs stimulated with PM_2.5_ compared with cells stimulated with vehicle (Figure 3B). Organotypic skin cultures were also stimulated with PM_2.5_ for 7 days and then stained for AHR. PM_2.5_-treated cell cultures had nuclear AHR localization (Figure 3C). A significant increase in AHR staining intensity was observed in organotypic skin treated with PM_2.5_ compared with skin treated with vehicle (*P* < 0.01) (Figure 3D). These findings indicate that PM_2.5_ induces AHR activation in keratinocytes.

### PM_2.5_ induction of TNF-α and inhibition of FLG in keratinocyte cultures is AHR dependent.

**Figure 4:**
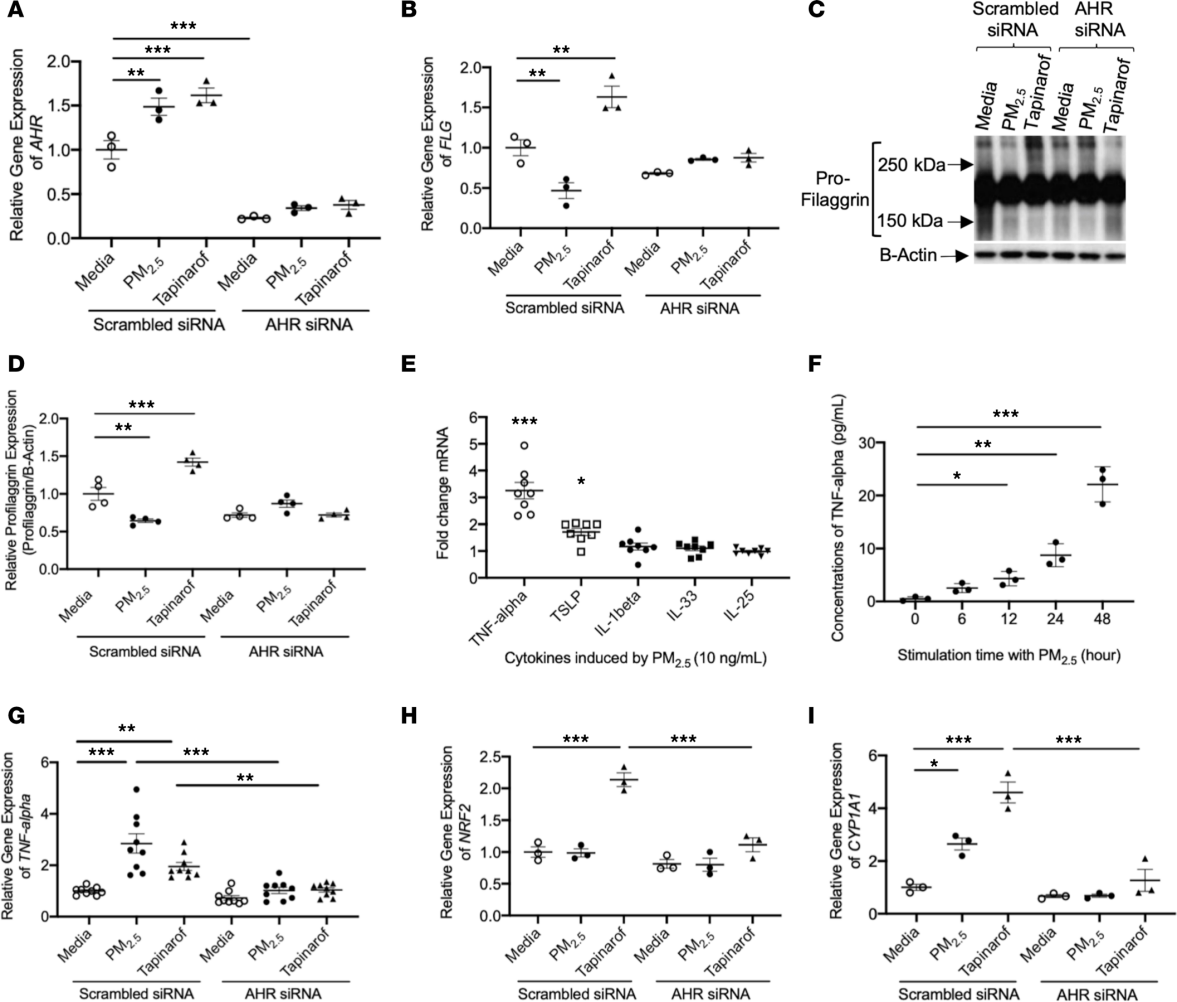
PM_2.5_ modulates expressions of FLG and TNF-α in human primary keratinocytes. Gene expressions of *AHR* (**A**), *FLG* (**B**), *TNFA* (**G**), *NRF2* (**H**), and *CYP1A1* (**I**) were examined in cultured HEKs using real-time RT-PCR. *n* = 3–9 per group. (**C** and **D**) Protein expression of FLG was evaluated in cultured HEKs using Western blotting. *n* = 4 per group. (**E**) Gene expressions of keratinocyte-derived cytokines were examined in cultured HEKs treated with PM_2.5_ (10 ng/mL) using real-time RT-PCR. *n* = 8 per group. (**F**) Protein levels of TNF-α were evaluated in culture media using ELISA. Data are representative of 3 independent experimental repetitions. The data are shown as the mean ± SEM. *P < 0.05, **P < 0.01, ***P < 0.001 by 1-way ANOVA with Tukey-Kramer post hoc test.

We further examined whether PM_2.5_ suppression of FLG expression is AHR dependent. HEKs were transfected with scrambled siRNA or *AHR* siRNA. This was followed by treatment with PM_2.5_ or tapinarof, which is known as an AHR agonist (36). Gene expression of *AHR* was significantly (*P* < 0.001) inhibited in cells transfected with *AHR* siRNA, as compared with cells transfected with scrambled siRNA (Figure 4A). Additionally, *AHR* gene expression was significantly upregulated by PM_2.5_ (*P* < 0.01) or tapinarof (*P* < 0.001) in cells transfected with scrambled siRNA but was not modulated in cells transfected with *AHR* siRNA (Figure 4A). Gene expressions of *FLG* (Figure 4B) and *IVL* (Supplemental Figure 2B) were not significantly affected by AHR siRNA, but *LOR* gene expression was significantly (*P* < 0.01) inhibited in cells transfected with AHR siRNA, as compared with cells transfected with scrambled siRNA (Supplemental Figure 2A). In HEKs transfected with scrambled siRNA, gene expression of *FLG* was significantly (*P* < 0.01) downregulated in cells treated with PM_2.5_ and significantly (*P* < 0.01) upregulated in cells treated with tapinarof as compared with cells treated with media alone (Figure 4B). However, *FLG* expression was not modulated by PM_2.5_ or tapinarof in cells transfected with *AHR* siRNA (Figure 4B). This finding was confirmed by Western blot (Figure 4, C and D). This suggests that PM_2.5_ inhibition of FLG is AHR dependent.

Inhibition of FLG expression by keratinocyte-derived cytokines including TNF-α (37), thymic stromal lymphopoietin (TSLP) (38), IL-1β (39), IL-33 (40), and IL-25 (41) has been documented. Hence, we further investigated whether PM_2.5_ modulates the expression of these cytokines. As depicted in Figure 4E, the gene expression of *TNFA* was profoundly induced by PM_2.5_. Moreover, TNF-α protein levels were significantly (*P* < 0.05) increased in HEK culture supernatants after 12-hour treatment with PM_2.5_ (Figure 4F). Additionally, PM_2.5_ significantly (*P* < 0.05) induced gene expression of *TNFA* after 6-hour stimulation and significantly (*P* < 0.001) inhibited gene expression of *FLG* after 24-hour stimulation in HEKs (Supplemental Figure 3). *TSLP* gene was modestly induced, but other keratinocyte-derived cytokines were not significantly changed by PM_2.5_ (Figure 4E).

We confirmed that in HEKs transfected with scrambled siRNA, gene expression of *TNFA* was significantly upregulated after PM_2.5_ (*P* < 0.001) or tapinarof (*P* < 0.01) treatment as compared with cells treated with media alone (Figure 4G). In contrast, *TNFA* expression was not induced by PM_2.5_ or tapinarof in cells transfected with *AHR* siRNA (Figure 4G), indicating that PM_2.5_- and tapinarof-induced TNF-α expression in keratinocytes is AHR dependent. Additionally, nuclear factor erythroid 2-related factor (NRF2) expression was evaluated because it was previously reported to be induced by AHR agonists such as tapinarof and was associated with FLG upregulation by this naturally derived AHR agonist (36, 42–44). In HEKs transfected with scrambled siRNA, gene expression of *NRF2* was not modulated by PM_2.5_ but significantly (*P* < 0.001) upregulated in the cells treated with tapinarof (Figure 4H). *NRF2* gene was not significantly induced by PM_2.5_ or tapinarof in the cells transfected with *AHR* siRNA (Figure 4H). Gene expressions of cytochrome P450, family 1, subfamily A, polypeptide 1 (*CYP1A1*), and glutathione-*S*-transferase Mu 1 (*GSTM1*) were also evaluated because they are induced by AHR ligands and play key roles in metabolizing PAHs (36, 45). Gene expression of *CYP1A1* was significantly induced by both PM_2.5_ (*P* < 0.05) and tapinarof (*P* < 0.001) in HEKs transfected with scrambled siRNA (Figure 4I), but *GSTM1* gene expression was only significantly (*P* < 0.01) induced by tapinarof in HEKs transfected with scrambled siRNA (Supplemental Figure 2C). The induction of *CYP1A1* by PM_2.5_ and tapinarof and the induction of *GSTM1* by tapinarof were shown to be AHR dependent, as they were not induced by corresponding treatments in cells transfected with *AHR* siRNA. Furthermore, the lack of *GSTM1* induction by PM_2.5_ may result in prolonged detrimental effects of PM_2.5_ in the skin.

### PM_2.5_-induced TNF-α inhibits FLG through the MAPK/c-JNK pathway.

**Figure 5:**
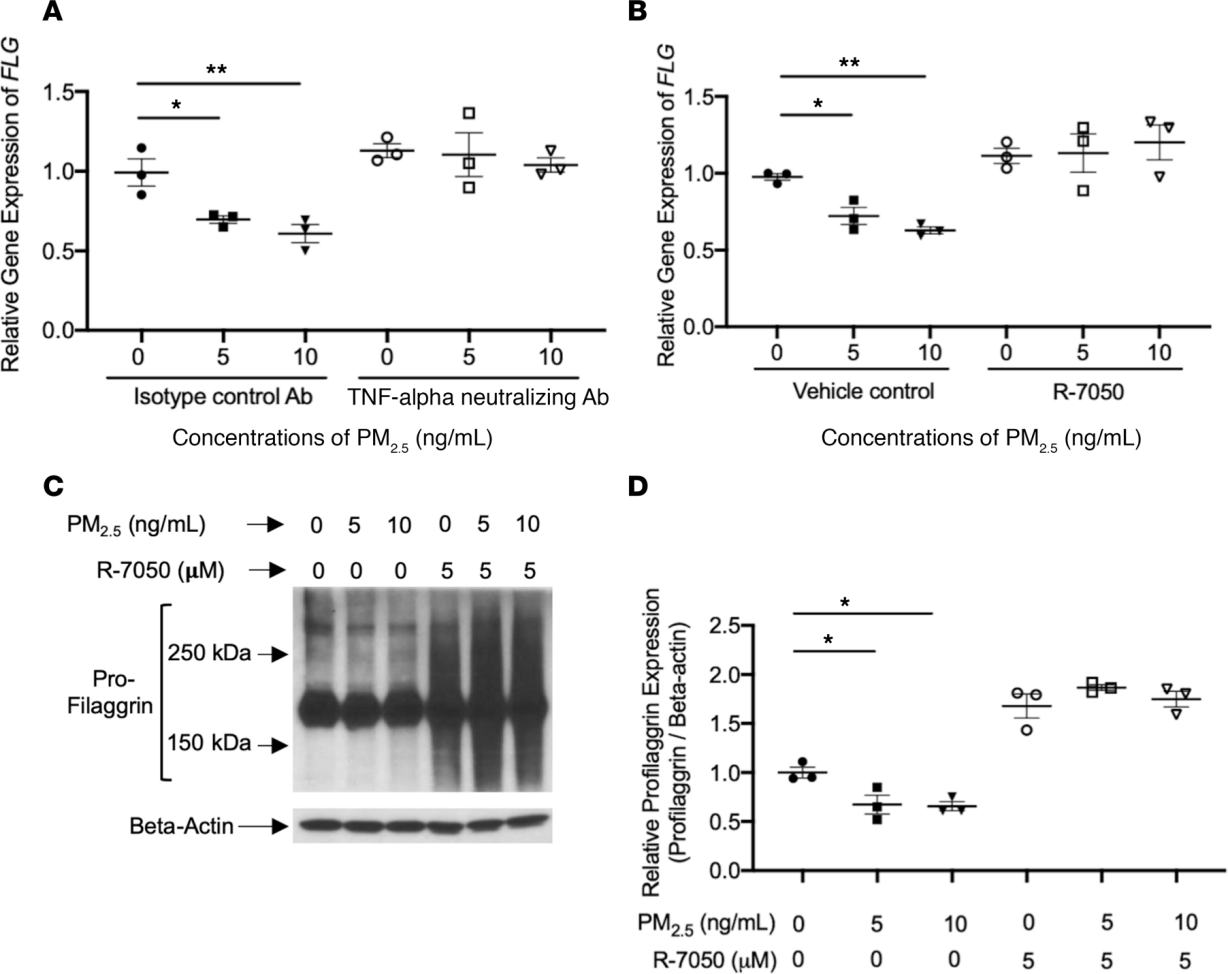
PM_2.5_-induced TNF-α inhibits FLG expression in human primary keratinocytes. Gene expression of *FLG* (**A** and **B**) was examined in cultured HEKs using real-time RT-PCR. Protein expression of FLG (**C** and **D**) was evaluated using Western blotting. Data are representative of 3 independent experimental repetitions. The data are shown as the mean ± SEM. *n* = 3 per group. *P < 0.05, **P < 0.01 by 1-way ANOVA with Tukey-Kramer post hoc test.

To determine the role of TNF-α in the regulation of FLG in PM_2.5_-treated keratinocyte cultures, HEKs were differentiated and preincubated with TNF-α neutralizing Ab (0.1 μg/mL), an isotype control Ab (0.1 μg/mL), vehicle control, or R-7050 (a TNF-α receptor type 1 inhibitor) for 24 hours, followed by stimulation with various concentrations of PM_2.5_ for an additional 2 days. *FLG* gene expression was significantly inhibited by PM_2.5_ at concentrations as low as 5 ng/mL in cells preincubated with the isotype control Ab (*P* < 0.05, Figure 5A) or vehicle control (*P* < 0.05, Figure 5B). In contrast, *FLG* gene expression was not modulated by PM_2.5_ in cells preincubated with the TNF-α neutralizing Ab (Figure 5A) or R-7050 (Figure 5B). This finding was confirmed at protein levels using Western blotting (Figure 5, C and D). Therefore, PM_2.5_-mediated inhibition of FLG is regulated by PM_2.5_-induced TNF-α.

**Figure 6:**
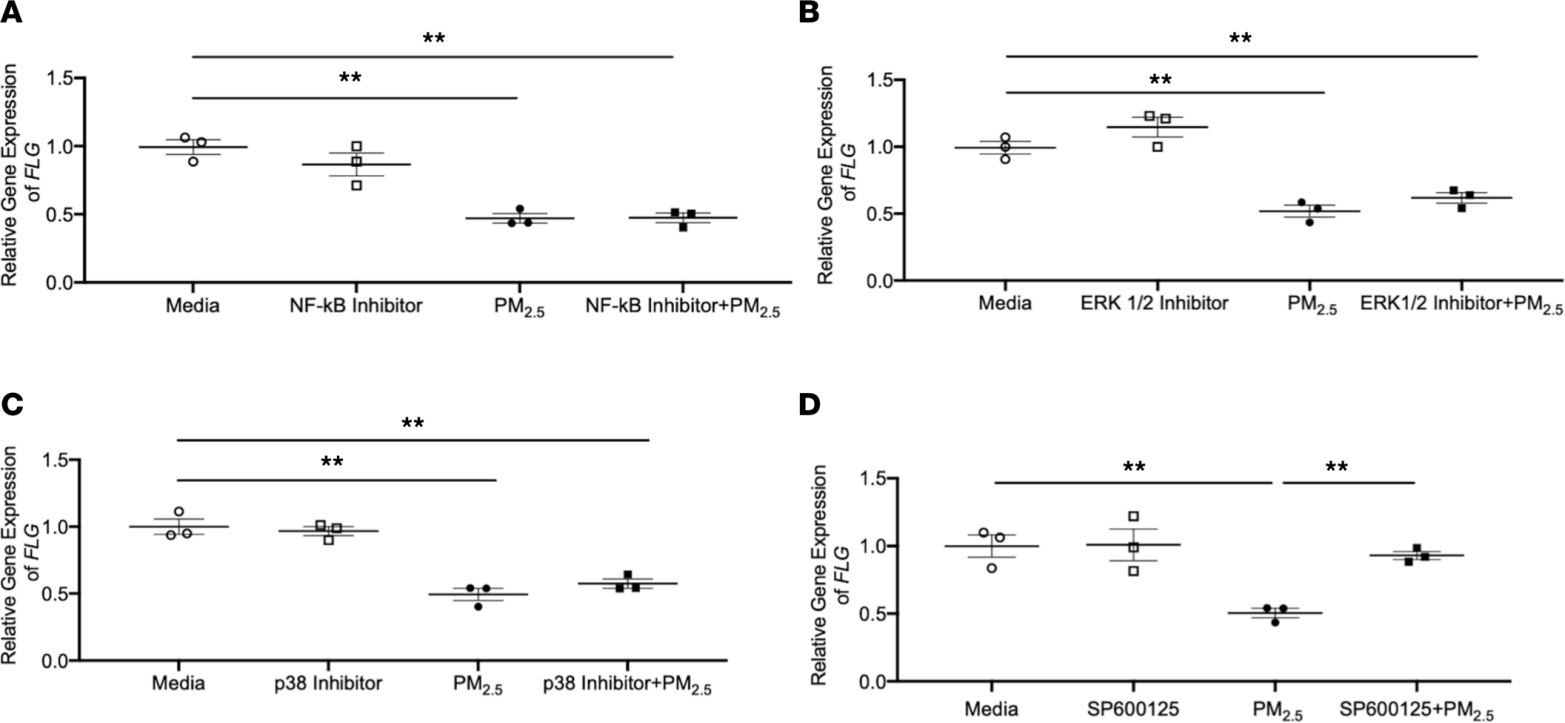
PM_2.5_ inhibits FLG through the MAPK/c-JNK pathway. Gene expression of *FLG* was examined using real-time RT-PCR in cultured HEKs stimulated with PM_2.5_ or combinations of each inhibitor and PM_2.5_. (**A**) NF-κB inhibitor. (**B**) ERK1/2 inhibitor. (**C**) P38 inhibitor. (**D**) SP600125 inhibitor. Data are representative of 3 independent experimental repetitions. The data are shown as the mean ± SEM. *n* = 3 per group. **P < 0.01 by 1-way ANOVA with Tukey-Kramer post hoc test.

We further studied the effects of downstream targets of TNF-α signaling on the regulation of FLG by PM_2.5_. HEKs were preincubated with an NF-κB inhibitor (10 nM) or MAPK inhibitors such as ERK1/2 inhibitor (5 μM), p38 inhibitor (5 μM), or c-JNK inhibitor SP600125 (0.5 μM) for 24 hours. The doses of inhibitors used in this study were previously proved to be nontoxic to the keratinocytes (37). Then the cells were stimulated with either PM_2.5_ (10 ng/mL) or a combination of each inhibitor and PM_2.5_ (10 ng/mL) for an additional 2 days. Gene expression of *FLG* was significantly decreased in the cells treated with the combinations of PM_2.5_ and NF-κB inhibitor (*P* < 0.01, Figure 6A), ERK1/2 inhibitor (*P* < 0.01, Figure 6B), or p38 inhibitor (*P* < 0.01, Figure 6C) as compared with the cells treated with media alone. However, *FLG* gene expression was not significantly inhibited in the cells treated with the combination of PM_2.5_ and SP600125 (Figure 6D), indicating that PM_2.5_ inhibits FLG via c-JNK. This finding is consistent with our previous observation that TNF-α downregulates FLG expression through the MAPK/c-JNK pathway (37).

### PM_2.5_ inhibits FLG by inducing TNF-α and increases TEWL in murine skin.

**Figure 7:**
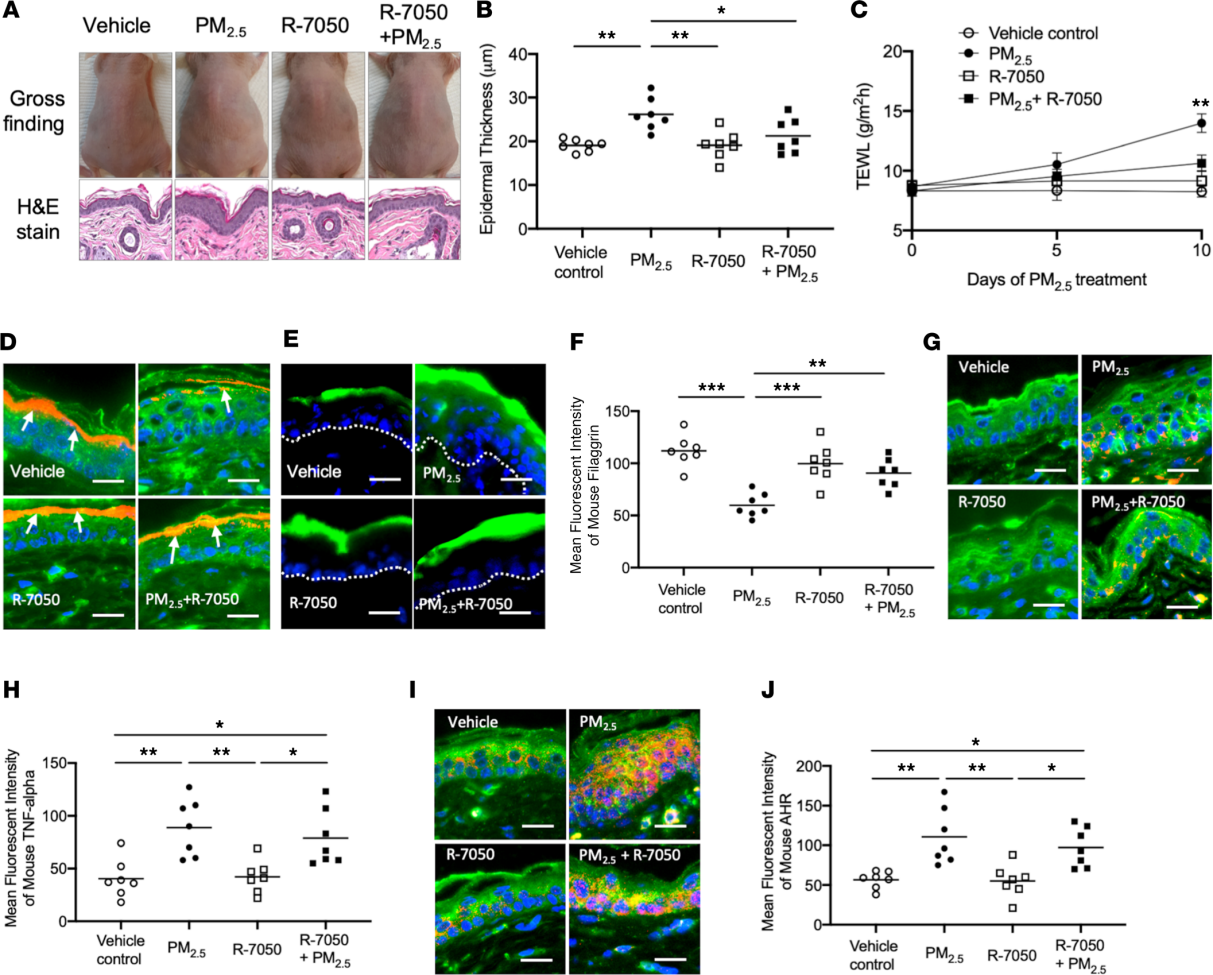
PM_2.5_ inhibits FLG and causes skin barrier dysfunction in murine skin. Hairless mice were treated with a vehicle, PM_2.5_, R-7050, or a combination of PM_2.5_ and R-7050 on the back of each mouse twice daily for 10 days. FITC-dextran was applied to the left side of the back of each mouse for 60 minutes on day 10 and demonstrated enhanced barrier penetration of the PM_2.5_-treated skin. (**A**) Skin appearance and H&E staining (original magnification, ×100) of the skin biopsy samples in the study groups. Epidermal thickness (**B**) and TEWL (**C**) were evaluated and illustrated increased epidermal thickness and TEWL in PM_2.5_-exposed skin. (**D**) The penetration of FITC-dextran is enhanced in PM_2.5_-treated skin and is attenuated by TNF-α inhibitors. Protein expressions of FLG (**E** and **F**), TNF-α (**G** and **H**), and AHR (**I** and **J**) were evaluated using immunofluorescence staining. Arrows point to FLG staining (red). The dotted line represents a border between epidermis and dermis. Wheat germ agglutinin–conjugated FITC (green) was used to stain the cytoskeleton. Nuclei were visualized with DAPI (blue). Data are representative of 2 independent experiments. The data are shown as the mean ± SEM. Each point indicates individual mice, *n* = 7 mice per group. Scale bar: 25 μm. *P < 0.05, **P < 0.01, ***P < 0.001 by 1-way ANOVA with Tukey-Kramer post hoc test.

Our in vitro experiments have demonstrated that PM_2.5_ inhibits FLG expression in keratinocytes and provided evidence for the involvement of the AHR/TNF-α pathway in this process. To confirm these effects of PM_2.5_ in vivo, vehicle control (0.2% DMSO in PBS), PM_2.5_ (100 ng/mL), R-7050 (5 μM), or a combination of PM_2.5_ (100 ng/mL) and R-7050 (5 μM) was applied on the backs of hairless mice twice daily for 10 days. No skin lesions, such as ulcers or inflammatory lesions, were noted in any mice after 10 days of treatment (Figure 7A). However, the thickness of epidermis in H&E staining was significantly increased (*P* < 0.01) in mouse skin treated with PM_2.5_ compared with skin treated with vehicle, R-7050, or the combination of PM_2.5_ and R-7050 (Figure 7, A and B). The TEWL was not significantly different between study groups on days 0 and 5. However, TEWL on day 10 was significantly (*P* < 0.01) increased in mice treated with PM_2.5_ compared with mice treated with vehicle control, R-7050, or the combination of PM_2.5_ and R-7050 (Figure 7C). These data suggest that PM_2.5_ causes skin barrier dysfunction in treated mice and that R-7050 attenuates PM_2.5_-mediated skin barrier dysfunction. In support of this observation, we also found that the penetration of FITC-dextran into epidermis was higher in skin treated with PM_2.5_ than skin treated with vehicle control, R-7050, or the combination of PM_2.5_ and R-7050 (Figure 7D).

As shown in Figure 7, E and F, the staining intensity of FLG was significantly (*P* < 0.001) decreased in the skin of mice treated with PM_2.5_ as compared with the skin treated with vehicle control. On the contrary, the staining intensity of FLG was significantly (*P* < 0.01) higher in the skin of mice treated with the combination of PM_2.5_ and R-7050 as compared with skin treated with PM_2.5_ alone (Figure 7, E and F), suggesting that blocking of TNF-α receptor attenuates PM_2.5_-mediated inhibition of FLG. Indeed, the staining intensity of TNF-α was significantly increased in the skin of mice treated with PM_2.5_ (*P* < 0.01) and the combination of PM_2.5_ and R-7050 (*P* < 0.05) as compared with the skin treated with vehicle or R-7050 (Figure 7, G and H), indicating that R-7050 does not change TNF-α levels but inhibits TNF-α signaling by blocking interaction with the TNF-α receptor. Furthermore, PM_2.5_ induced nuclear localization of AHR in the skin cells (Figure 7I), and the staining intensity of AHR was significantly (*P* < 0.01) increased in the skin of mice treated with PM_2.5_ as compared with the skin treated with vehicle or R-7050 (Figure 7J). These findings confirm that PM_2.5_ induces nuclear translocation of AHR in the cells.

## Discussion

PM is a ubiquitous atmospheric aerosol and a complex mixture of various components, which include PAHs, nitrate, sulfate, ammonium, elemental carbon, heavy metals, and so on (1, 6). PM is primarily derived from either natural or anthropogenic sources, such as forest fires, sea salts, biologic materials (i.e., pollen, endotoxin, fungi, bacteria), biomass combustion, vehicle exhaust, and power plants (1, 6). It is also generated secondarily from precursors emitted in the air, like sulfur oxides, nitrogen oxides, volatile organic compounds, and ammonia (1, 6). Cigarette smoking is another documented source of PM (46, 47). Additionally, wildfire events from mountainous areas such as the northwestern region of the United States significantly increase the levels of PM_2.5_ (48, 49). The airborne concentration of PM_2.5_ in ambient conditions is about 50~400 μg/m^3^ in major cities worldwide, but it reaches up to 800 μg/m^3^ in big cities (2, 25, 26, 50).

In the present study, we report that skin FLG levels in the same subjects decreased after they moved from Denver, USA, to Seoul, South Korea within the same season, with skin samples assessed only 1–2 months apart. We speculated that these changes could be driven by air pollution, as the skin FDP levels were decreased when the study subjects moved to a high-PM area (Seoul) from the low-PM area (Denver). However, the levels of *trans*-UCA were not changed in normal healthy subjects, although total-UCA was changed in the present study. Therefore, it seems *trans*-UCA is less affected by environmental factors such as PM. This finding is consistent with a recent report that cold and dry air do not change the levels of *trans*-UCA (51). These data have limitations because other environmental factors, such as humidity, temperature, and UV radiation, are not the same in Seoul and Denver and may have also contributed to changes in FDP. In this study, we provide experimental evidence that PM_2.5_ exposure can influence skin barrier function and likely is one of the contributing factors that influenced skin barrier function in our study subjects. We focused on the relationship of PM_2.5_ and FLG, a critical component of the epidermal skin barrier (14, 19, 20), because it has been suggested that PM_2.5_ is strongly associated with the development of inflammatory skin diseases (21, 22), and FLG is a key epidermal barrier protein in maintaining skin barrier function (20, 35).

In this study, we demonstrate significant inhibition of FLG expression in keratinocyte cultures, organotypic skin cultures, and the skin of the animals exposed in vivo to PM_2.5_. This observation is consistent with previous reports (27, 29). However, this is the first study that examined the effects of PM_2.5_ on skin both in vitro and in vivo. Importantly, the doses of PM_2.5_ used in our experiments were not directly cytotoxic and reflect the doses of PM_2.5_ that are achievable in highly polluted urban areas such as Seoul, Korea, and Beijing, China. In contrast, earlier studies examined the effects of PM in the HaCat tumor keratinocyte cell line and human keratinocytes, using doses that far exceed those in the environment. We found that these PM_2.5_ doses were cytotoxic to primary keratinocytes in the current study (27–29).

We demonstrate for the first time to our knowledge that PM_2.5_-induced TNF-α plays a crucial role in PM_2.5_-mediated inhibition of FLG. PM_2.5_-mediated induction of TNF-α is an earlier event with subsequent inhibition of FLG by TNF-α occurring after. We propose that keratinocytes and neutrophils could be the main sources of TNF-α after PM_2.5_ exposure. Previous studies reported that PM_2.5_ induces the recruitment of neutrophils (52, 53), which produces TNF-α (54). Additionally, we demonstrated that *FLG* and *LOR* were inhibited by PM_2.5_, which did not affect *IVL*. This finding implies that PM_2.5_-induced TNF-α is associated with PM_2.5_-mediated inhibition of epidermal barrier proteins because we previously reported that TNF-α inhibits *FLG* and *LOR* but not *IVL* (37). Moreover, PM_2.5_ did not inhibit *FLG* in both keratinocytes and mouse skin preincubated with TNF-α neutralizing Ab, R-7050, or SP600125 (c-JNK inhibitor). These findings strongly suggest that PM_2.5_-mediated inhibition of *FLG* is via the action of PM_2.5_-induced TNF-α. Here we documented that PM_2.5_ regulation of TNF-α is AHR dependent in vitro and in vivo. Importantly, we also provide evidence that TNF-α neutralizing Ab or a small molecule inhibitor of the TNF-α receptor, R-7050, can prevent detrimental effects of PM_2.5_ exposure on skin barrier function.

AHR, a ligand-dependent transcription factor, is activated by multiple compounds that can induce either epidermal differentiation or oxidative stress (43, 44). It has been reported that tapinarof, an AHR agonist, upregulates FLG expression in keratinocytes (36). In the current study, we demonstrated that both tapinarof and PM_2.5_ induced TNF-α via AHR but modulated FLG expression in opposite ways. To understand this discrepancy, we further investigated the expression of NRF2 in keratinocytes, a potent suppressor of ROS (36, 55), in response to these AHR ligands. Interestingly, *NRF2* was not induced by PM_2.5_ but was robustly induced by tapinarof in an AHR-dependent manner. These findings suggest that PM_2.5_-induced TNF-α inhibits FLG, but the effects of tapinarof-induced TNF-α on FLG could be negated by the action of tapinarof-induced NRF2. This mechanism is supported by previous studies. Activation of NRF2 by AHR ligands such as coal tar abolished IL-4/IL-13–mediated inhibition of epidermal barrier proteins such as FLG and LOR (42, 56). Additionally, activation of the AHR/NRF2 pathway by ketoconazole showed cytoprotective effects and inhibition of TNF-α–induced production of ROS (55). Therefore, we demonstrate a ligand-dependent dual action of AHR using 2 agonists.

AHR is essential in maintaining normal skin barrier function and cutaneous homeostasis (57, 58). The components of the PM_2.5_ might be resistant to degradation, thus, resulting in persistent AHR activation. Indeed, mice with a constitutively active form of AHR in keratinocytes (*AHR*-CA mouse) have been shown to develop an AD-like skin disease by induction of artemin, which causes pruritus and skin scratches followed subsequently by skin inflammation and barrier dysfunction (12, 59). Additionally, activation of the AHR/artemin axis in *AHR*^fl/fl^ mice by air pollutants induces nerve hyperinnervation and alloknesis, which causes scratch and exacerbation of AD skin (12). In contrast to the toxic effects of persistently activated AHR, AHR itself is beneficial for the skin barrier, as the AHR-deficient mice show skin inflammation, epidermal barrier dysfunction, and skin dysbiosis (57, 58). Thus, both *AHR*-CA and AHR-deficient mice have detrimental effects on the skin. However, these mice are not a viable condition of human skin and do not reflect human skin that is exposed to air pollutants such as PM. Human skin exposure to PM could be different from *AHR*-CA or AHR-deficient mice. In our current study, we focused on PM_2.5_-mediated skin barrier function directly because skin barrier dysfunction is considered an initial step of the development of AD (14, 19). Additionally, AHR was activated in normal human keratinocytes and nonatopic mice skin with physiologic concentrations of PM_2.5_ that are achievable in the polluted urban area. Therefore, we report that PM_2.5_ causes skin barrier dysfunction in normal and nonatopic skin in naturally occurring environments.

It has been suggested that maintaining normal skin barrier function is critical for the prevention and control of allergic diseases such as AD and food allergy (14, 16, 60–62). It is also well known that FLG deficiency and increased TEWL are associated with skin barrier dysfunction and increased allergen sensitization (20, 35, 63–65). In the current study, we demonstrated that PM_2.5_-induced TNF-α causes FLG deficiency, increased TEWL, and enhanced penetration of FITC-dextran in both organotypic and mouse skin. We have also shown that *TSLP* expression was modestly increased by PM_2.5_ treatment in the current study. Our research team previously reported that increased TSLP expression in unaffected young infants was a good predictor of AD development in this Korean study cohort; however, no mechanism for the early TSLP induction in the skin was provided in that study (66). Our observation that PM_2.5_ exposure may increase TSLP production in the skin suggests that the increased skin TSLP expression in young infants could be associated with PM_2.5_ exposure in high-PM_2.5_ areas. It is noteworthy that the prevalence of AD in South Korea is nearly 30% compared with less than 20% in the United States (67, 68). We propose that air pollution and skin PM_2.5_ exposure may serve as one of the environmental factors that compromise skin barrier function and may promote allergen sensitization through the skin, thus further promoting incidence of allergic disorders worldwide.

Recently, Chan et al. have reported that TNF-α is highly expressed in Chinese AD skin compared with European AD skin (69). The concentrations of PM_2.5_ in Asian countries are higher than in European countries (https://aqicn.org) (25, 70, 71). Our present observation that PM_2.5_ induces TNF-α expression could further explain why TNF-α is higher in Asian AD skin. Additionally, our present findings may also account for the previous observation that the Asian AD phenotypes have features of psoriasis with increased Th17 polarization, which were linked to TNF-α exposure (72). Furthermore, the incidence and the prevalence of AD in Asian countries, such as China and Korea, where there are high-PM_2.5_ areas, are similar to those of European ancestry despite *FLG* mutations being more uncommon in Chinese and Korean ancestry (20, 67, 73, 74). Therefore, we hypothesize that PM_2.5_-associated FLG deficiency and skin barrier dysfunction play pivotal roles in the development of AD in Asian countries.

In summary, our present study demonstrates that PM_2.5_-induced TNF-α causes FLG deficiency in the skin, contributing to skin barrier dysfunction, and results in enhanced skin barrier penetration. We document that this process in skin keratinocytes is AHR dependent. Therefore, PM_2.5_ skin exposure may promote the development of allergic diseases by inducing FLG deficiency and skin barrier dysfunction, allowing antigen penetration through the skin. The study suggests that interventions that will prevent skin exposure to PM_2.5_ may be critical for protection from the detrimental effects of PM_2.5_ on skin barrier and potential allergic sensitization through the skin. Our in vivo animal model of PM_2.5_ exposure demonstrates that TNF-α receptor inhibitors can be used in vivo to protect from PM-induced skin barrier changes in PM-exposed mice and suggests that topical TNF receptor antagonists may be considered for skin protection from air pollution.

## Methods

### Preparation of PM_2.5_ and determination of PM_2.5_ concentration for experiments.

The PM_2.5_ were provided by the Seoul Metropolitan Government Research Institute of Public Health and Environment (Seoul, Korea). The air samplers were placed 10.3 m above the ground in the central area of Seoul. Twenty-four-hour integrated PM_2.5_ samples were collected on quartz filters (8 × 10 in., Tissuquartz 2500QAT-UP, Pall Life Sciences) by negative pressure using a high-volume sampler (model HV-1000F, Sibata Scientific Technology) at 1000 L/min flow rate during the winter season between December 2017 and January 2018. The quartz filters were precombusted in a muffle furnace at 600°C for 2 hours to remove any contaminants on the filters before sampling. The collected filters were then stored at –20°C until particle extraction was performed. For the experiments, the filter papers were cut into small pieces and suspended in PBS with 10% DMSO. The outdoor concentrations of PM_2.5_ in cities such as Seoul, Korea, and Beijing, China, reach up to 200 μg/m^3^ (about 0.2 ng/mL) and 800 μg/m^3^ (about 0.8 ng/mL), respectively (https://aqicn.org) (2, 25, 70, 75). PAHs penetrate into the epidermis easily (8–10) and are stable for 3~6 weeks in vivo (76). Additionally, PM_2.5_ can be accumulated in the skin because they remain in the skin up to 7 days (8, 10). Thus, the concentration of PM_2.5_ in the human skin is estimated to be higher than that of PM_2.5_ in outdoor air. Therefore, 1~10 ng/mL and 100 ng/mL of PM_2.5_ were used for our present in vitro and in vivo experiments, respectively. These doses were confirmed to be not cytotoxic using lactate dehydrogenase (LDH) release assay as described below.

### Collection and analysis of STS samples.

To examine the effects of air pollution in the skin, STS samples were collected from 5 subjects (4 normal, healthy subjects and 1 convalescent AD subject; mean age = 35 ± 7.4 years; 4 males and 1 female). All subjects had lived in Denver, Colorado, USA, for at least 1 month when initial STS samples were collected, and then STS samples were collected again from the same subjects after they moved to and stayed in Seoul, Korea, for at least 1 month. They received similar medical care at both locations and utilized similar skincare routines during the study period. All samples were collected during the same winter season. The concentration of PM_2.5_ is typically higher in Seoul (100~180 μg/m^3^) and lower in Denver (25~35 μg/m^3^) during winter (https://aqicn.org). A total of 10 consecutive D-Squame tape discs (CuDerm) were applied to the volar side of the forearms of subjects as previously described (77). STS samples were processed and analyzed for FDP, *cis*/*trans*-UCA, and PCA using an LC-ESI-MS/MS as previously described (17).

### Cell culture.

HEKs (Life Technologies, Thermo Fisher Scientific) were used to examine the effects of PM_2.5_ exposure in vitro. HEKs were derived from neonatal foreskin and were grown in serum-free EpiLife cell culture medium (Life Technologies, Thermo Fisher Scientific) containing 0.06 mmol/L CaCl_2_, 1% human keratinocyte growth supplement S7 (Life Technologies, Thermo Fisher Scientific), and 1% gentamicin/amphotericin. To investigate the effects of the PM_2.5_ on the expression of FLG, AHR, TNF-α, and other keratinocyte-derived cytokines, HEKs were differentiated in the presence of 1.3 mmol/L of CaCl_2_ for 3 days. Then cells were stimulated with PM_2.5_ (5 and 10 ng/mL), IL-4 (10 ng/mL), and IL-13 (10 ng/mL) or IFN-γ (10 ng/mL) for various time periods up to 48 hours.

To examine whether PM_2.5_ induces nuclear translocation of AHR, HEKs were seeded into the 8-well Nunc Lab-Tek II Chamber Slides (Thermo Fisher Scientific) and stimulated with PM_2.5_ (1 ng/mL) for 24 hours. To examine whether PM_2.5_ inhibition of FLG is TNF-α dependent, the cells were preincubated with the isotype control Ab (0.1 μg/mL) (R&D Systems, Bio-Techne), TNF-α neutralizing Ab (0.1 μg/mL) (R&D Systems, Bio-Techne), vehicle control (medium with 0.1% DMSO), or the TNF-α receptor 1 inhibitor, R-7050 (5 μM) (Tocris), for 24 hours. Then the cells were stimulated with PM_2.5_ (5 and 10 ng/mL) for an additional 2 or 4 days. HEKs were preincubated with NF-κB activation inhibitor (10 nM, MilliporeSigma) or MAPK inhibitors (MilliporeSigma), including ERK1/2 inhibitor (5 μM), p38 inhibitor (5 μM), or c-JNK inhibitor SP600125 (0.5 μM), for 24 hours, followed by cell stimulation with PM_2.5_ (10 ng/mL) or combinations of each inhibitor and PM_2.5_ (10 ng/mL) for an additional 2 days. EpiLife cell culture medium with 0.1% DMSO was used as a vehicle control for all in vitro experiments, as PM_2.5_ was diluted with EpiLife cell culture medium with 0.1% DMSO.

### LDH assay.

HEKs were plated at 2 × 10^5^ cells per well in a 24-well plate and differentiated in the presence of 1.3 mmol/L of CaCl_2_ for 3 days. To examine cell toxicity of the PM_2.5_, cells were treated with various concentrations of PM_2.5_ for 48 hours, and LDH release was then determined using the CytoTox-One Homogeneous Membrane Integrity Assay (Promega) according to the manufacturer’s instructions. Fluorescence was measured on a DTX880 Multimode Detector (Beckman Coulter) with an excitation wavelength of 560 nm and an emission wavelength of 590 nm. Samples were tested in triplicate and fluorescence results were normalized by subtracting a PBS blank and compared with the keratinocyte LDH release in response to treatment with 1% Triton X-100 solution (maximum LDH release).

### RNA preparation and real-time RT-PCR.

RNeasy Mini Kits (Qiagen) were used according to the manufacturer’s protocol to isolate RNA from keratinocytes. RNA was reverse-transcribed into cDNA using SuperScript VILO MasterMix according to the manufacturer’s protocol (Life Technologies, Thermo Fisher Scientific). cDNA was analyzed by real-time RT-PCR using an ABI Prism 7300 sequence detector (Applied Biosystems, Thermo Fisher Scientific). Primers and probes for 18S RNA, *AHR*, *FLG*, *LOR*, *IVL*, keratin-1, desmocollin-1, corneodesmosin, claudin-1, *TNFA*, *TSLP*, *IL1B*, *IL*33, *IL25*, *NRF2*, and *CYP1A1* were purchased from Applied Biosystems, Thermo Fisher Scientific.

### Western blot.

For Western blot, 15 μg of total proteins extracted from HEKs were separated on 4%–20% SDS-polyacrylamide gel (Bio-Rad) and transfected to the cellulose membrane. The blots were blocked with Super Block (Scyteck Laboratories) and incubated with primary Abs. The Abs against FLG (catalog sc-66192, Santa Cruz Biotechnology) and β-actin (catalog SAB3500350, MilliporeSigma) were used as primary Abs. The densitometry of the detected protein bands was calculated using ImageJ software, version 1.49 (NIH).

### TNF-α protein measurement.

TNF-α protein expression levels in cell culture supernatant were measured using an ELISA kit (R&D Systems, Bio-Techne) per the manufacturer’s instructions. Expression levels of TNF-α were determined by comparison to a standard curve generated by serial dilution of a manufacturer-provided standard.

### Organotypic skin culture.

To produce organotypic skin culture using HEKs, NIH 3T3 J2 murine embryonic fibroblasts (ATCC, CCL-92) and culture insert (BD Biosciences) were used. Murine fibroblasts were cultured in DMEM containing 4.5 g/L glucose (Corning). A mixture of fibroblasts and rat tail type I collagen (Corning) were plated onto the culture inserts as a dermal equivalent. HEKs (2 × 10^6^/culture insert) were then plated on top of the dermal equivalent, air-lifted after 1 day, and cultured for 7 days in DMEM containing 1% of gentamicin/amphotericin and growth factors such as adenine, insulin, apo-transferrin, and triiodothyronine (Corning). The air-liquid interface organotypic skin cultures were stimulated with a vehicle control (culture medium with 0.1% DMSO) or PM_2.5_ (1 ng/mL) applied to the surface of the air-liquid interface culture for an additional 7 days, and then the skin was fixed with 4% buffered formalin for immunostaining.

### Small interfering RNA silencing experiments.

Control and *AHR* siRNA duplexes were purchased from Horizon Discovery. HEKs were plated in 6-well or 24-well plates the day before transfection. The 5 pmol siRNA duplexes were transfected into HEKs using Lipofectamine 2000 (Life Technologies, Thermo Fisher Scientific) according to the manufacturer’s instructions. The following day, the cells were differentiated in the presence of 1.3 mmol/L of CaCl_2_ for 3 days, and the cells were then incubated with medium, PM_2.5_ (10 ng/mL), or an AHR agonist, tapinarof (0.1 μM, MedChemExpress), for an additional 2 or 4 days for RT-PCR or Western blotting, respectively.

### Murine model to examine the effects of PM_2.5_ in the skin.

Hairless mice (Crl: SKH1-*Hrhr*, female, 12 weeks old, strain 477, Charles River Laboratories) were used for animal experiments. To investigate whether PM_2.5_ inhibits FLG and whether this process is TNF-α dependent, mice (*n* = 7 per group) were treated with a vehicle (PBS with 0.2% DMSO), PM_2.5_ (100 ng/mL), R-7050 (10 μM), or combination of PM_2.5_ (100 ng/mL) and R-7050 (10 μM). Stimulants were applied on the back of each mouse twice daily for 10 days. R-7050 was applied 1 hour before PM_2.5_ treatment each time. FITC-dextran (MilliporeSigma) was applied for 60 minutes on the left side of the back of each mouse on day 10 to examine the penetration of FITC-dextran into the skin. Skin punch biopsies (4 mm) were collected and stored at –80°C immediately. Frozen sections were prepared to assess FITC-dextran penetration into the skin. All mice were euthanized on day 10, and 2 skin biopsies (4 mm) were also obtained from each animal. The skin biopsies were submerged immediately into either Tri-Reagent (Molecular Research Center, Inc.) or 10% buffered formalin for real-time RT-PCR and immunostaining, respectively.

### Immunofluorescence staining.

Cells grown in chamber slides, organotypic skin sections, or mouse skin sections were fixed and blocked. Slides were then stained with Abs against AHR (catalog orb35828, Biorbyt), human FLG (catalog ab81468, Abcam), mouse FLG (catalog 905804, BioLegend), and TNF-α (catalog 11948, Cell Signaling Technology). Nuclei were visualized with DAPI, and wheat germ agglutinin–conjugated FITC was used to stain the cytoskeleton. Frozen slide sections were generated from mouse skin biopsies treated with FITC-dextran using the Tissue-Tek OCT compound (Scigen Scientific) and Cryomold Intermediate (Thermo Fisher Scientific). The slides were visualized with fluorescence microscopy (Leica). Images were collected at original magnification ×400, and the levels of MFI were measured with Slidebook 6.0 (Intelligent Imaging Innovations).

### Measurement of TEWL.

TEWL was measured using a device from GPower Inc. in both organotypic skin cultures and mouse skin. To measure TEWL in organotypic skin, culture medium was removed from the culture chamber and insert. Then, the excess medium from the organotypic skin was allowed to air-dry for 10 minutes. TEWL from the organotypic skin cultures was measured every other day from day 8 to 14 of culture. TEWL from mouse skin was measured on days 0, 5, and 10 on the back of each mouse. All TEWL measurements from both organotypic and mouse skin were collected in triplicate, and the average values were used for graphs.

### Statistics.

Statistical analysis was conducted using GraphPad Prism, version 8.4.2. In cases where 2 groups were compared, data were analyzed using a paired or unpaired 2-tailed Student’s *t* test. Statistical differences between 3 or more groups were determined by using 1-way ANOVA, and significant differences were determined by a Tukey-Kramer post hoc test. All error bars represent mean ± SEM. Statistical significance was defined as *P* < 0.05.

### Study approval.

STS sample collection was approved by the Institutional Review Boards at both National Jewish Health, Denver, Colorado, USA (IRB no. HS-3146), and Samsung Medical Center, Seoul, Korea (IRB no. SMC-2018-03-041). All subjects gave written informed consent prior to participation in the study. Animal experiments were approved by the Institutional Animal Care and Use Committee of Samsung Medical Center (IRB no. SMC-2020-03-25001).

## Author contributions

BEK, DYML, and KA conceived the study. BEK, JK, and EG designed and performed experiments. EB and IB performed mass spectrometry and analyzed the data. JL, KAV, and CFH performed real-time RT-PCR and ELISA. SH and NRK performed Western blotting. BEK and KAV generated organotypic skin and performed immunostaining. JK, JL, UHL, and SL assisted with skin tape sample collection and subject characterization. KA and HRY assisted with PM collection. BEK, JL, SH, NRK, and EJL performed animal experiments. BEK, JK, EG, DYML, and KA analyzed, interpreted, and synthesized data and wrote the manuscript. Co–first authors are listed based on alphabetical order.

## Supplementary Material

Supplemental data

## Version 1 01/26/2021

In-Press Preview

## Version 2 03/08/2021

Electronic publication

## References

[1] Guarnieri M, Balmes JR (2014). Outdoor air pollution and asthma. Lancet.

[2] Cooper DM, Loxham M (2019). Particulate matter and the airway epithelium: the special case of the underground?. Eur Respir Rev.

[3] Lelieveld J (2019). Cardiovascular disease burden from ambient air pollution in Europe reassessed using novel hazard ratio functions. Eur Heart J.

[4] Zhang P, Zhou X (2020). Health and economic impacts of particulate matter pollution on hospital admissions for mental disorders in Chengdu, Southwestern China. Sci Total Environ.

[5] Burnett R (2018). Global estimates of mortality associated with long-term exposure to outdoor fine particulate matter. Proc Natl Acad Sci U S A.

[6] Adams K (2015). Particulate matter components, sources, and health: systematic approaches to testing effects. J Air Waste Manag Assoc.

[7] Jin SP, et al. Urban particulate matter in air pollution penetrates into the barrier-disrupted skin and produces ROS-dependent cutaneous inflammatory response in vivo [published online April 30, 2018]. *J Dermatol Sci*. https://doi.org/10.1016/j.jdermsci.2018.04.015 10.1016/j.jdermsci.2018.04.015 29731195

[8] Ng KM (1992). Percutaneous absorption and metabolism of pyrene, benzo[a]pyrene, and di(2-ethylhexyl) phthalate: comparison of in vitro and in vivo results in the hairless guinea pig. Toxicol Appl Pharmacol.

[9] Kao J (1985). Skin penetration and metabolism of topically applied chemicals in six mammalian species, including man: an in vitro study with benzo[a]pyrene and testosterone. Toxicol Appl Pharmacol.

[10] Chu I (1996). Skin reservoir formation and bioavailability of dermally administered chemicals in hairless guinea pigs. Food Chem Toxicol.

[11] Kabashima K (2016). Linking air pollution to atopic dermatitis. Nat Immunol.

[12] Hidaka T (2017). The aryl hydrocarbon receptor AhR links atopic dermatitis and air pollution via induction of the neurotrophic factor artemin. Nat Immunol.

[13] Kabashima K (2019). The immunological anatomy of the skin. Nat Rev Immunol.

[14] Leung DYM (2020). Cutaneous barrier dysfunction in allergic diseases. J Allergy Clin Immunol.

[15] Ahn K (2020). Recent advances in atopic dermatitis. Curr Opin Immunol.

[16] Brough HA (2020). Epicutaneous sensitization in the development of food allergy: what is the evidence and how can this be prevented?. Allergy.

[17] Leung DYM (2019). The nonlesional skin surface distinguishes atopic dermatitis with food allergy as a unique endotype. Sci Transl Med.

[18] Kim BE, Leung DYM (2018). Significance of skin barrier dysfunction in atopic dermatitis. Allergy Asthma Immunol Res.

[19] Goleva E (2019). Epithelial barrier repair and prevention of allergy. J Clin Invest.

[20] Irvine AD (2011). Filaggrin mutations associated with skin and allergic diseases. N Engl J Med.

[21] Ngoc LTN (2017). Systematic review and meta-analysis of human skin diseases due to particulate matter. Int J Environ Res Public Health.

[22] Huls A (2019). Nonatopic eczema in elderly women: effect of air pollution and genes. J Allergy Clin Immunol.

[23] Tang KT (2017). Adult atopic dermatitis and exposure to air pollutants-a nationwide population-based study. Ann Allergy Asthma Immunol.

[24] Kim J (2013). Symptoms of atopic dermatitis are influenced by outdoor air pollution. J Allergy Clin Immunol.

[25] Kim YM (2018). The effects of particulate matter on atopic dermatitis symptoms are influenced by weather type: application of spatial synoptic classification (SSC). Int J Hyg Environ Health.

[26] Oh I (2018). Association between particulate matter concentration and symptoms of atopic dermatitis in children living in an industrial urban area of South Korea. Environ Res.

[27] Lee CW (2016). Urban particulate matter down-regulates filaggrin via COX2 expression/PGE2 production leading to skin barrier dysfunction. Sci Rep.

[28] Li Q (2017). Effects of ambient fine particles PM_2.5_ on human HaCaT cells. Int J Environ Res Public Health.

[29] Hieda DS (2020). Air particulate matter induces skin barrier dysfunction and water transport alteration on a reconstructed human epidermis model. J Invest Dermatol.

[30] Kang M (2020). Recent occurrence of PAHs and n-Alkanes in PM2.5 in Seoul, Korea and characteristics of their sources and toxicity. Int J Environ Res Public Health.

[31] Dutton SJ (2010). Temporal patterns in daily measurements of inorganic and organic speciated PM_2.5_ in Denver. Atmos Environ (1994).

[32] Xie M (2012). Intra-urban spatial variability of PM_2.5_-bound carbonaceous components. Atmos Environ (1994).

[33] Kezic S (2011). Levels of filaggrin degradation products are influenced by both filaggrin genotype and atopic dermatitis severity. Allergy.

[34] Howell MD (2007). Cytokine modulation of atopic dermatitis filaggrin skin expression. J Allergy Clin Immunol.

[35] Brown SJ, McLean WH (2012). One remarkable molecule: filaggrin. J Invest Dermatol.

[36] Smith SH (2017). Tapinarof is a natural AhR agonist that resolves skin inflammation in mice and humans. J Invest Dermatol.

[37] Kim BE (2011). TNF-α downregulates filaggrin and loricrin through c-Jun N-terminal kinase: role for TNF-α antagonists to improve skin barrier. J Invest Dermatol.

[38] Kim JH (2015). Thymic stromal lymphopoietin downregulates filaggrin expression by signal transducer and activator of transcription 3 (STAT3) and extracellular signal-regulated kinase (ERK) phosphorylation in keratinocytes. J Allergy Clin Immunol.

[39] Bernard M (2017). IL-1β induces thymic stromal lymphopoietin and an atopic dermatitis-like phenotype in reconstructed healthy human epidermis. J Pathol.

[40] Seltmann J (2015). IL-33 impacts on the skin barrier by downregulating the expression of filaggrin. J Allergy Clin Immunol.

[41] Kim BE (2013). IL-25 enhances HSV-1 replication by inhibiting filaggrin expression, and acts synergistically with Th2 cytokines to enhance HSV-1 replication. J Invest Dermatol.

[42] Furue M (2020). Regulation of filaggrin, loricrin, and involucrin by IL-4, IL-13, IL-17A, IL-22, AHR, and NRF2: pathogenic implications in atopic dermatitis. Int J Mol Sci.

[43] Haarmann-Stemmann T (2012). The AhR-Nrf2 pathway in keratinocytes: on the road to chemoprevention?. J Invest Dermatol.

[44] Furue M (2019). Aryl hydrocarbon receptor in atopic dermatitis and psoriasis. Int J Mol Sci.

[45] Nan HM (2001). Effects of occupation, lifestyle and genetic polymorphisms of CYP1A1, CYP2E1, GSTM1 and GSTT1 on urinary 1-hydroxypyrene and 2-naphthol concentrations. Carcinogenesis.

[46] Gurley ES (2013). Seasonal concentrations and determinants of indoor particulate matter in a low-income community in Dhaka, Bangladesh. Environ Res.

[47] Kantor R (2016). Association of atopic dermatitis with smoking: a systematic review and meta-analysis. J Am Acad Dermatol.

[48] McClure CD, Jaffe DA (2018). US particulate matter air quality improves except in wildfire-prone areas. Proc Natl Acad Sci U S A.

[49] Marsha A, Larkin NK (2019). A statistical model for predicting PM_2.5_ for the western United States. J Air Waste Manag Assoc.

[50] Zhu YY (2019). [Concentration characteristics and assessment of model-predicted results of PM2.5 in the Beijing-Tianjin-Hebei region in autumn and winter]. Huan Jing Ke Xue.

[51] Jung M (2019). Exposure to cold airflow alters skin pH and epidermal filaggrin degradation products in children with atopic dermatitis. Allergol Int.

[52] Miyake T (2018). Endocytosis of particulate matter induces cytokine production by neutrophil via Toll-like receptor 4. Int Immunopharmacol.

[53] Jeong S (2019). PM2.5 Exposure in the respiratory system induces distinct inflammatory signaling in the lung and the liver of mice. J Immunol Res.

[54] Grivennikov SI (2005). Distinct and nonredundant in vivo functions of TNF produced by t cells and macrophages/neutrophils: protective and deleterious effects. Immunity.

[55] Tsuji G (2012). Identification of ketoconazole as an AhR-Nrf2 activator in cultured human keratinocytes: the basis of its anti-inflammatory effect. J Invest Dermatol.

[56] van den Bogaard EH (2013). Coal tar induces AHR-dependent skin barrier repair in atopic dermatitis. J Clin Invest.

[57] Haas K (2016). Aryl hydrocarbon receptor in keratinocytes is essential for murine skin barrier integrity. J Invest Dermatol.

[58] Di Meglio P (2014). Activation of the aryl hydrocarbon receptor dampens the severity of inflammatory skin conditions. Immunity.

[59] Tauchi M (2005). Constitutive expression of aryl hydrocarbon receptor in keratinocytes causes inflammatory skin lesions. Mol Cell Biol.

[60] Simpson EL (2014). Emollient enhancement of the skin barrier from birth offers effective atopic dermatitis prevention. J Allergy Clin Immunol.

[61] Horimukai K (2014). Application of moisturizer to neonates prevents development of atopic dermatitis. J Allergy Clin Immunol.

[62] Lowe AJ (2018). A randomized trial of a barrier lipid replacement strategy for the prevention of atopic dermatitis and allergic sensitization: the PEBBLES pilot study. Br J Dermatol.

[63] Nikolovski J (2008). Barrier function and water-holding and transport properties of infant stratum corneum are different from adult and continue to develop through the first year of life. J Invest Dermatol.

[64] Boralevi F (2008). Epicutaneous aeroallergen sensitization in atopic dermatitis infants - determining the role of epidermal barrier impairment. Allergy.

[65] Flohr C (2010). Filaggrin loss-of-function mutations are associated with early-onset eczema, eczema severity and transepidermal water loss at 3 months of age. Br J Dermatol.

[66] Kim J (2016). Epidermal thymic stromal lymphopoietin predicts the development of atopic dermatitis during infancy. J Allergy Clin Immunol.

[67] Ahn K (2016). The prevalence of atopic dermatitis in Korean children. Allergy Asthma Immunol Res.

[68] McKenzie C, Silverberg JI (2019). The prevalence and persistence of atopic dermatitis in urban United States children. Ann Allergy Asthma Immunol.

[69] Chan TC (2018). Atopic dermatitis in Chinese patients shows T_H_2/T_H_17 skewing with psoriasiform features. J Allergy Clin Immunol.

[70] Cheng NL (2016). [Concentration characteristics of PM2.5 in Beijing during two red alert periods]. Huan Jing Ke Xue.

[71] Fang GC (2018). Review of total suspended particles (TSP) and PM_2.5_ concentration variations in Asia during the years of 1998–2015. Environ Geochem Health.

[72] Noda S (2015). The Asian atopic dermatitis phenotype combines features of atopic dermatitis and psoriasis with increased TH17 polarization. J Allergy Clin Immunol.

[73] Yu HS (2013). Mutations in the filaggrin are predisposing factor in Korean children with atopic dermatitis. Allergy Asthma Immunol Res.

[74] O’Regan GM (2008). Filaggrin in atopic dermatitis. J Allergy Clin Immunol.

[75] Lv B (2017). Understanding the rising phase of the PM_2.5_ concentration evolution in large China cities. Sci Rep.

[76] Wolff RK (1989). Effects of adsorption of benzo[a]pyrene onto carbon black particles on levels of DNA adducts in lungs of rats exposed by inhalation. Toxicol Appl Pharmacol.

[77] Kim BE (2019). Side-by-side comparison of skin biopsies and skin tape stripping highlights abnormal stratum corneum in atopic dermatitis. J Invest Dermatol.

