# Supplementary material for: Particulate matter causes skin barrier dysfunction
Source: JCI Insight. 2021 Mar 8;6(5):e145185. doi: 10.1172/jci.insight.145185 (PMC8021104; doi:10.1172/jci.insight.145185)
Supplement: Supplemental data [file jciinsight-6-145185-s126.pdf]

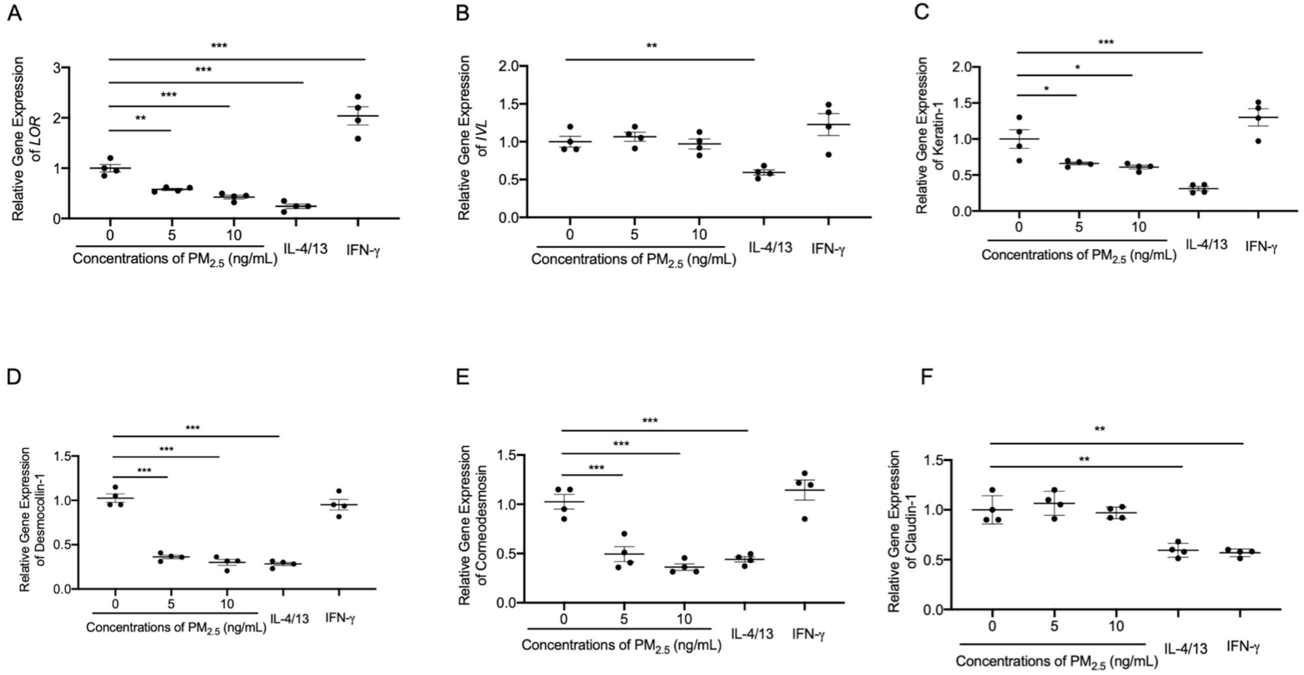

Supplementary Figure 1. $PM_{2.5}$ modulates the epidermal barrier proteins in human primary keratinocytes.

HEKs were differentiated for 3 days and then the cells were stimulated with various concentrations of $PM_{2.5}$, Th2 cytokines (IL-4 and IL-13), or IFN-γ for an additional 48 h. Gene expressions of *LOR* (A), *IVL* (B), keratin-1 (C), desmocollin-1 (D), corneodesmosin (E), and claudin-1 (F) were evaluated using real-time RT-PCR. Data are representative of three independent experimental repetitions. The data are shown as the mean ± SEM. n = 3 per group. *P<0.05, **P<0.01, ***P<0.001 by one-way ANOVA with Tukey-Kramer host-hoc test.

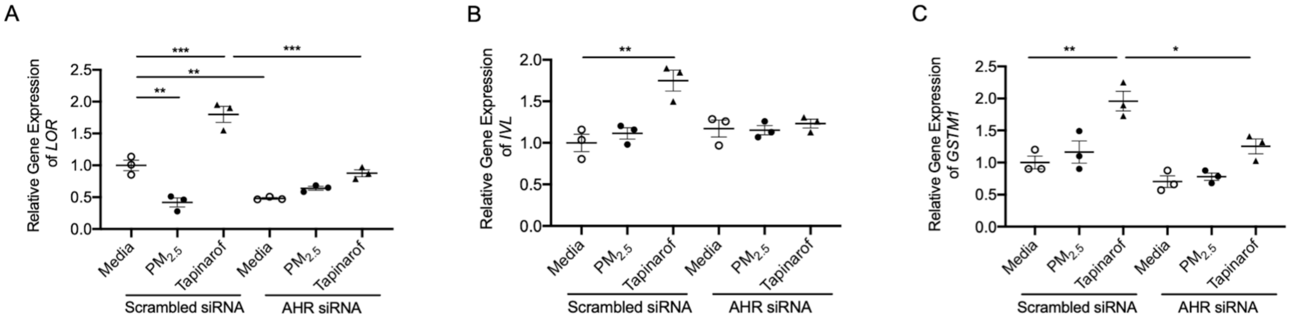

Supplementary Figure 2. $PM_{2.5}$ and tapinarof modulate gene expressions of epidermal barrier proteins and *GSTM1* in human primary keratinocytes.

Gene expressions of *LOR* (A), *IVL* (B), and *GSTM1* (C) were examined in cultured HEKs using real-time RT-PCR. n = 3 per group. Data are representative of three independent experimental repetitions. The data are shown as the mean ± SEM. **P*<0.05, ***p*<0.01, ****p*<0.001 by one-way ANOVA with Tukey-Kramer host-hoc test.

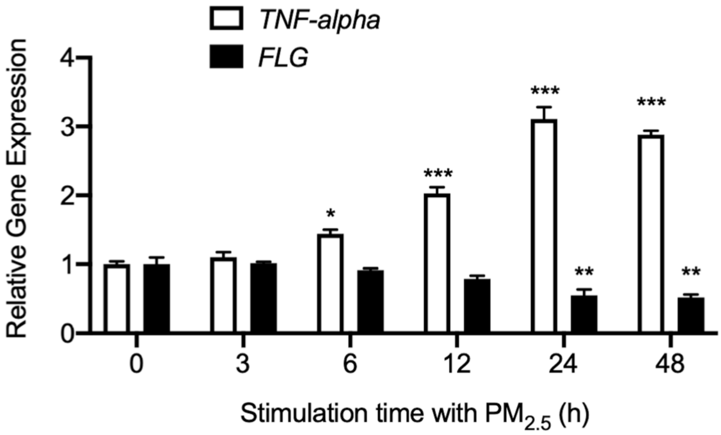

Supplementary Figure 3. $PM_{2.5}$ induces expression of TNF-alpha and inhibits FLG subsequently in human primary keratinocytes.

HEKs were differentiated for 3 days and then the cells were stimulated with $PM_{2.5}$ for various periods up to 48 h. Gene expressions of *TNF-alpha* and *FLG* were evaluated using real-time RT-PCR. Data are representative of three independent experimental repetitions. The data are shown as the mean ± SEM. n = 3 per group. *$P<0.05$, **$P<0.01$, ***$P<0.001$ by repeated measures ANOVA with Tukey-Kramer host-hoc test.
